# Enhanced adsorptive removal of rifampicin and tigecycline from single system using nano-ceria decorated biochar of mango seed kernel

**DOI:** 10.1016/j.heliyon.2023.e15802

**Published:** 2023-04-26

**Authors:** Marwa El-Azazy, Ahmed S. El-Shafie, Reem Al-Mulla, Siham S. Hassan, Hassan I. Nimir

**Affiliations:** Department of Chemistry and Earth Sciences, College of Arts and Sciences, Qatar University, Doha, 2713, Qatar

**Keywords:** Adsorption, Biochar, Antibiotics removal, Ceria nanoparticles, Selectivity, Regeneration

## Abstract

Pharmaceutically active compounds (PhACs) represent an emerging class of contaminants. With a potential to negatively impact human health and the ecosystem, existence of pharmaceuticals in the aquatic systems is becoming a worrying concern. Antibiotics is a major class of PhACs and their existence in wastewater signifies a health risk on the long run. With the purpose of competently removing antibiotics from wastewater, cost-effective, and copiously available waste-derived adsorbents were structured. In this study, mango seeds kernel (MSK), both as a pristine biochar (Py–MSK) and as a nano-ceria-laden (Ce–Py–MSK) were applied for the remediation of rifampicin (RIFM) and tigecycline (TIGC). To save time and resources, adsorption experiments were managed using a multivariate-based scheme executing the fractional factorial design (FrFD). Percentage removal (%R) of both antibiotics was exploited in terms of four variables: pH, adsorbent dosage, initial drug concentration, and contact time. Preliminary experiments showed that Ce–Py–MSK has higher adsorption efficiency for both RIFM and TIGC compared to Py–MSK. The %R was 92.36% for RIFM compared to 90.13% for TIGC. With the purpose of comprehending the adsorption process, structural elucidation of both sorbents was performed using FT-IR, SEM, TEM, EDX, and XRD analyses which confirmed the decoration of the adsorbent surface with the nano-ceria. BET analysis revealed that Ce–Py–MSK has a higher surface area (33.83 m^2^/g) contrasted to the Py–MSK (24.72 m^2^/g). Isotherm parameters revealed that Freundlich model best fit Ce–Py–MSK–drug interactions. A maximum adsorption capacity (*q*_*m*_) of 102.25 and 49.28 mg/g was attained for RIFM and TIGC, respectively. Adsorption kinetics for both drugs conformed well with both pseudo-second order (PSO) and Elovich models. This study, therefore, has established the suitability of Ce–Py–MSK as a green, sustainable, cost-effective, selective, and efficient adsorbent for the treatment of pharmaceutical wastewater.

## Graphical abstract

**Figure:**
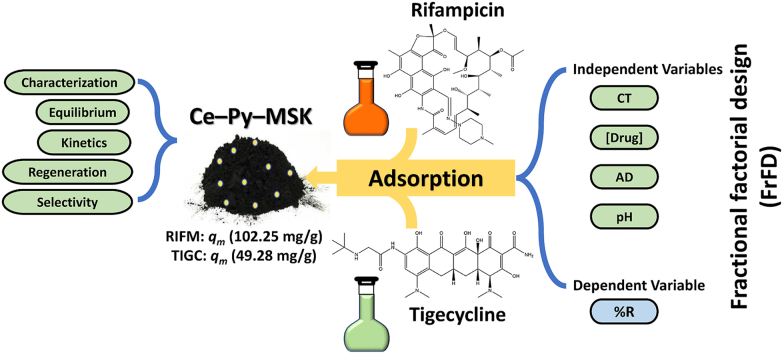

## Abbreviations

PhACs: Pharmaceutically active compounds
MSK: Mango seeds kernel
Py–MSK: Pyrolyzed mango seed kernel (pristine biochar)
Ce–Py–MSK: Nano-ceria-laden biochar
RIFM: Rifampicin
TIGC: Tigecycline
FrFD: Fractional factorial design
%R: Percentage removal
FT-IR: Fourier-transform infrared spectroscopy
SEM: Scanning electron microscopy
TEM: Transmission electron microscopy
EDX: Energy-dispersive X-ray spectrometer
XRD: X-ray diffraction pattern
BET: Brunauer- Emmett-Teller analysis
*q*_*m*_: Maximum adsorption capacity (mg/g)
PSO: Pseudo-second order model
FDA: US Food and Drug Administration
AD: Sorbent dosage
CT: Contact time
DW: Deionized water
C_0_ and C_e_: Initial and equilibrium concentrations (ppm)
*q*_*e*_: Adsorption capacity (mg/g)
W: Mass of the adsorbent (g)
V: Volume of RIFM and TIGC solutions (L)
%Er: % Relative error
Obs: Observed responses
Pred: Predicted responses
TGA: Thermogravimetric analysis
PSD: Particle size distribution
*k*, *p*: Number of variables, fraction index
Ct Pt: Central point
SD: Standard deviation
*d*: Individual desirability function
D-R: Dubinin-Radushkevich model
*K*_*L*_: Langmuir equilibrium coefficient
*R*_*L*_: The separation factor
K_F_, 1/n: Freundlich constants
A_T_, b_T_: Temkin constants
R: Universal gas constant
q_s_: Saturation capacity
β: Activity coefficient
ε: Calculated Polanyi potential
K_1_, K_2_: Adsorption rate constant
α, β: Elovich constants
K_I_: Intraparticle diffusion rate constant
C: Thickness of the boundary layer

## 1 Introduction

**Scheme 1:**
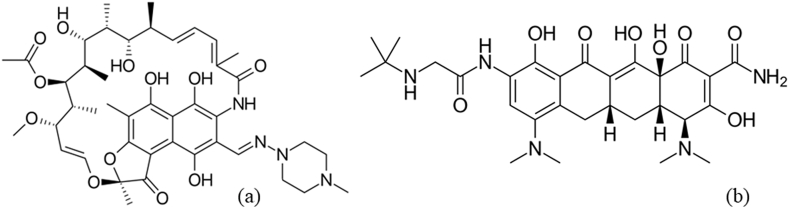
(A) Rifampicin [RIFM] and (b) Tigecycline [TIGC].

With the rapid development in the pharmaceutical industry laid back with an improved perception of life quality standards, consumption of pharmaceutically active compounds (PhACs) is becoming a daily routine. Consequently, it is of no wonder that PhACs are increasingly detected in aquatic environments posing serious health and ecological risks [[1], [2], [3]]. Generally, antibiotics–a major class of PhACs–act as secondary metabolites, that are capable of inhibiting the microbial growth [[4], [5], [6]]. Rifampicin (RIFM) and tigecycline (TIGC), Scheme 1, are broad-spectrum antibiotics. The impact of their existence in aquatic environments could be also described as ‘broad-spectrum’, that is because they are: bio-cumulative, toxic, and cannot be eliminated by the traditional wastewater remediation techniques [[5], [6], [7], [8]].

RIFM possesses antibacterial activity against an assortment of infective microorganisms. The range of activities of RIFM includes tuberculosis, leprosy, meningitis, and recently as a repurposed drug to treat COVID-19 [[9], [10], [11], [12], [13], [14], [15], [16]]. As one of the frontline therapeutics against leprosy, the treatment protocol using RIFM usually extends for 6–9 months. This prolonged consumption implies increased excretion and hence excessive release into aquatic environments [[17], [18], [19], [20]]. Tigecycline (TIGC), a tetracycline antibiotic, is commonly used to treat infections caused by multidrug-resilient bacteria [15,[21], [22], [23]]. TIGC is largely removed unmodified in feces (59%) and urine (22%) [24]. Following the occurrence of unexplained deaths linked to administration of TIGC, the FDA has released a black-box warning for TIGC [25]. It is noteworthy to mention that both drugs are usually administered without prescription in North Africa.

As shown in Scheme 1, the functional groups encountered in both drugs explain their water solubility, an issue that augments their environmental risks, and fosters the ability of most microorganisms to carry antibiotics resistant genes [26]. The resilient genes together with the antibiotics’ residues are recognized as emerging pollutants [27]. Therefore, finding suitable approaches for the remediation of RIFM and TIGC is a must.

Several studies in literature portray the widespread application of waste-derived materials for environmental remediation [[28], [29], [30], [31], [32]]. Biochar derived from waste materials demonstrates significant features such as a functional surface, high surface area and porosity. Such attributes together with the liability for nanocomposite formulation render biochar-based materials excellent adsorbents for various contaminants.

Remediation of RIFM and TIGC was widely reported in literature with foci being centered on RIFM. Efforts included photocatalytic degradation [10,14], Fenton-oxidation alone, coupled with electro-oxidation, and UV-A homogeneous photo-Fenton [18,33,34]. Energy costs and the generation of byproducts limits the applicability of photocatalysis. Consumption of chemicals, the high costs, the difficulty of catalyst regeneration and the low degradation efficiency are the major obstacles that hamper the applicability of Fenton-processes on a large scale. Adsorption, on the contrary, is a fast, cost–efficient, and easy–to–assemble approach. Moreover, the quality of the treated effluents is high with no toxic secondary products. Sorptive removal of RIFM and TIGC using biomasses and biochar-based materials was reported [[9], [10], [11], [12], [13],^15^,^23^,^35^].

The world production of mangoes (*Mangifera indica* L.) was ∼56 million tons in 2019. The seed represents 35–55% of the mango fruit and is made up of a hard coat that encloses the kernel whose content varies from 45.7 to 72.8%. The kernel is commonly made up of crude protein, oil, ash, crude fiber, and carbohydrates [[36], [37], [38]]. Tons of mango stones are thrown annually and could represent a burden on the ecosystem if not recycled. The depollution efforts using MSK were mainly devoted for the heavy metals and dyes, with no single report for the deployment of MSK for the remediation of the pharmaceutical wastewater [[39], [40], [41], [42], [43]]. On the other hand, decoration of biochars with metal oxides renders them more efficient biosorbents for remediation of wastewater [[44], [45], [46]]. Cerium (IV) oxide has attracted the attentions thanks to its low cost, absence of the environmental footprint, and high stability. Existence of ceria in a multivalent state facilitates metal complexation with other materials. Literature shows that nano-ceria-tailored sorbents have displayed excellent performance for the wastewater remediation purposes [[47], [48], [49], [50]].

To date, existing efforts still lack the complete control of the variables impacting the remediation processes. Thinking of adsorption in specific, managing the adsorbent performance and efficiency as well as sustaining the process greenness could be attained via exerting control on the influencing variables: pH, sorbent dosage (AD), contact time (CT), and pollutant concentration. Such control helps diminishing the consumption of chemicals and resources. The approach we are using herein involves the implementation of a multivariate scheme; fractional factorial design (FrFD) [51,52]. Table 1 shows an overview of the utilization of biomass and biochar-derived materials for the removal of RIFM and TIGC. As could be comprehended, most of the reported approaches utilized a univariate scheme, with all the cons such a scheme could bring. Even with the reported multivariate-based approaches, either the achieved %R was lower compared to the current approach, or adsorbent preparation involved the consumption of large quantity of alkaline reagents impacting the final cost, and the ecotoxicity. Selectivity is still a concern in this case.

**Table 1:** Reported methods used for sorptive removal of rifampicin (RIFM) and tigecycline (TIGC).

Pollutant	Adsorbent	Preparation	Removal scenario	Surface area (m^2^/g)	*q*_*m*_ (mg/g)	%R	Ref
RIFM	Nano CeO_2_ decorated mango seed kernel	Microemulsion method	Multivariate based	33.83	RIFM:102.25	92.36%	This study
TIGC					TIGC: 49.28	90.13%	
Rifampicin	Activated carbon of cocoa shells	Shells were washed, dried at 50 °C for 24 h, then grinded	Univariate based	*NS	26.66	80%	[9]
Rifampicin	ZnCr layered double hydroxide (LDH)	Co-precipitation method	Univariate based	LDH:1.2	*NS	Adsorption was negligible and degradation was achieved via sono-photocatalysis	[10]
	ZnCr LDH/carbon nanotube (CNT)			CNT: 5.0			
	ZnCr LDH/Biochar (BC)			BC: 7.0			
Rifampicin	Calcined *Mytella falcata* shells	Calcination at 700 °C for 5 h	Univariate based	*NS	9.731	96.0%	[11]
Rifampicin	Sisal–Fe/Zn bio-nanocomposites	Co-precipitation method	Univariate based	*NS	40.00	96.0%	[12]
Rifampicin	Chitosan derived from mud-crab shells	Deacetylation of chitin isolated from the shells	Univariate based	6.57 ± 0.05	66.91	74.2%	[13]
Tigecycline	Olive stone biochar – decorated with Co_3_O_4_	Microemulsion method	Multivariate based	39.85	25.94	75.48%	[15]
Tigecycline	Magnetic biochar	Co-precipitation method	Multivariate based	86.06	57.14	99.91%	[23]
Tigecycline	*Chlorella pyrenoidosa*	Cultivation, centrifugation, then filtration	Univariate based	*NS	*NS	90%	[35]

*NS: Not stated.

The current approach involves designing a model adsorbent via recycling of MSK. The nano-ceria decorated sorbent (Ce–Py–MSK) will be tested and its performance will be optimized. To the best of our information, this is the first approach entailing the use of biochar of MSK whether pristine or ceria-decorated via a multivariate-controlled scheme for the depollution of antibiotics. Moreover, the selectivity of the current adsorbent towards different pollutants will be tested. Understanding the adsorption process of each drug on either adsorbent will be tackled employing various isotherm and kinetic models.

## 2 Experimental

### 2.1 Materials and reagents

All drugs including, RIFM, TIGC, amantadine, acetaminophen, sofosbuvir, acyclovir, and ivermectin were products of Biosynth® Carbosynth Ltd. (UK). Cerium (III) nitrate hexahydrate was product of Fluka–Garantie (Switzerland). Ammonia solution (26%) was product of Honeywell Riedel-*de*Haën AG (Germany). Oleylamine, ammonia solution (25%), rose bengal, fuchsine, methylene blue, ibuprofen sodium, and rest of chemicals were commodities of Sigma–Aldrich (USA). Deionized water (DW) (Millipore-Q water supply, 18.2 MΩ cm) was used thru this study. The ripened mango fruits were obtained locally, and the seeds were separated from the fruit. The hard seed coat was then removed, and the kernel was taken out (MSK). Stock solutions (100 ppm) of RIFM and TIGC and their following dilutions were prepared in DW and used for the following batch sorption studies. An aqueous solution of either HCl or NaOH (0.1 M) was utilized to fine-tune the pH to the requested value.

### 2.2 Equipment and software

A Memmert oven was used for drying the MSK (GmbH + Co. KG, Germany). To get the biochar of the MSK, a Thermolyne furnace (USA) was operated at 500 °C. Separation of the adsorbent from the contaminant was accomplished employing a Thermo Fisher Scientific centrifuge (USA). Drug–adsorbents suspensions were filtered applying non-sterile 0.45 μm nylon Millex syringe filters. To determine the absorbance of the filtrates, an Agilent diode-array UV–Vis spectrophotometer (USA) was used. Thermal stability of Py–MSK and Ce–Py–MSK was explored using thermogravimetric analysis (TGA, PerkinElmer-TGA400). The manifestation of functionalities on the surfaces of Py–MSK and Ce–Py–MSK was explored using FT-IR spectroscopy (PerkinElmer, USA). To examine the morphology of the produced sorbents, scanning electron microscopy (SEM, Thermo Scientific, USA) including energy-dispersive X-ray spectrometer (EDX) was operated. The later was employed to reconnoiter the elemental composition of Py–MSK and Ce–Py–MSK. Raman analysis (Thermo Scientific, USA) was utilized to examine the graphitic structure and the existence of nano-ceria on the surface following the decoration process. A transmission electron microscope (TEM) was utilized to investigate the microstructure of the prepared sorbents. Surface properties were assessed using a Micrometrics ASAP2020 instrument. Computations of surface area and the pore volume on the surface of Py–MSK and Ce–Py–MSK were conducted using the 77 K isotherms and the Brunauer-Emmett-Teller (BET) equation, as well as t-plots and implementing the Barrett-Joyner-Halenda (BJH) equation. The X-ray diffraction pattern (XRD) was determined using X'Pert-Pro MPD (PANalytical Co., the Netherlands) operated using Cu Kα X-ray source (λ = 1.540598 Å). The analysis was performed over a 3 h range of 5–90° (2θ). Minitab®19 program procured from Minitab Inc. (USA) was exploited to create the FrFD and evaluate the data acquired from the sorption investigations.

### 2.3 Preparation of the biochar (Py–MSK)

The MSK was initially washed 10 times with DW to get rid of any dirt and left to dry at 70 °C for 3 days. To obtain the biochar, clean and dry MSK was burnt in sealed crucibles at 500 °C for 1 h. The product, MSK, was portioned into two halves; the first was labelled as (Py–MSK) and was sealed in tightly closed bottles for the subsequent applications. The second portion was kept for decoration with nano-ceria as will be explained in the next sections.

### 2.4 Preparation of the nanoparticles (Ce–Py–MSK)

**Scheme 2:**
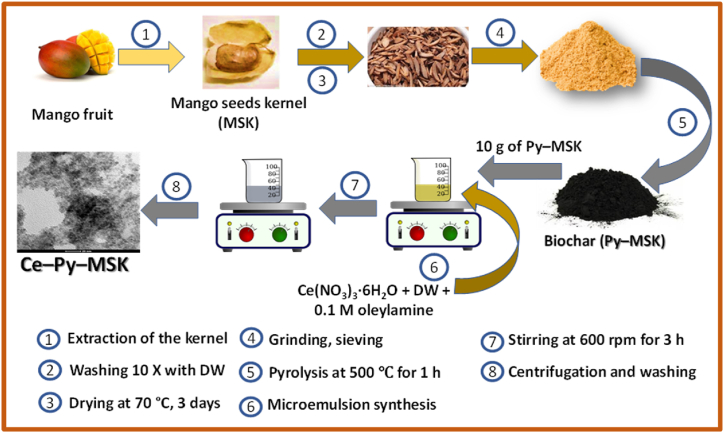
Schematic illustration of the preparation of Py–MSK and Ce–Py–MSK.

Py–MSK was decorated with nano-ceria using the microemulsion-based approach with slight changes in the reported procedure [53]. In this itinerary, Ce–Py–MSK was synthesized by dissolving 3.099 g of Ce(NO_3_)_3_·6H_2_O, keeping the ration of (Ce: Py–MSK) (w: w) as 1:10, in 300 mL of DW. With continuous stirring at a steady speed of 600 rpm, 10 g of Py–MSK were added followed by 100 mL of 0.1 M surfactant (oleylamine) prepared in n-propanol and the mixture was left for 3 h. Nano-ceria were precipitated at pH 12 b y adding drops of ammonia solution (26%). The product was washed 5 times using DW, left to dry at 70 °C overnight and the yield was marked as Ce–Py–MSK. A schematic representation of the detailed preparation of both adsorbents is portrayed in Scheme 2.

### 2.5 Evaluation of the point of zero charge (pH_PZC_)

pH_PZC_ was experimentally determined applying the pH drift method [54,55]. In this approach, seven solutions of 0.01 M sodium chloride were prepared. The pH in the range of 3.0–9.0 ± 0.2 (marked as pH_initial_) was attained using 0.1 M solutions from either HCl or NaOH. Mixtures containing 25 mL of the prepared solutions and 0.20 g of either Py–MSK or Ce–Py–MSK were prepared. The prepared mixtures were stirred for 48 h at a constant stirring speed of 150 rpm. Conclusively, the pH of the prepared solutions was determined, and the measured pH was marked as pH_final_. Unless otherwise specified, the measurements were performed in triplicate, and the results were presented as the mean value ± standard deviation (SD).

### 2.6 Application of the fractional factorial design (FrFD)

FrFD was operated to test the variables significance. Tested factors are shown in Table 2. The batch adsorption experimental set-up is displayed in Table 3. The FrFD involved 16 experimental trials comprising 8 central points (Ct Pt) and was carried out over 2 blocks. The response, %removal of RIFM and TIGC (%R), was assessed in terms of the four variables, Table 2. Eqs. (1), (2) were operated to calculate the experimental values of %R and the adsorption capacity, *q*_*e*_ (mg/g). The attained values of %R are revealed in Table 3. Predicted values shown in Table 3 were calculated using Minitab®19.

(1) $$\left(\%R\right)=\frac{C_{0}-C_{e}}{C_{0}}\;⨯\;100\%$$

(2) $$\left(q_{e},mg/g\right)=\frac{C_{0}-C_{e}}{W}\;V$$

In these two equations, C_0_ and C_e_ symbolize the initial and equilibrium concentrations (ppm), in that order, of RIFM and TIGC solutions, W the mass of the adsorbent (g), and V the volume of RIFM and TIGC solutions (L).

**Table 2:** Assessed effects and their levels.

Effect–Code	Unit	Low (−1)	Mid (0)	High (+1)
pH, (A)	pH unit	3.0	6.0	9.0
Adsorbent Dosage (AD), (B)	mg/13 mL	30	75	120
Contact Time (CT), (C)	Min	10	50	90
Initial [Drug], (D)	Ppm	20	60	100

**Table 3:** Obtained and expected values for the measured response: %R for RIFM and TIGC onto Ce–Py–MSK adsorbent and the %relative error (%Er).

Trial #	Effects				RIFM			TIGC		
	pH	AD	CT	[Drug]	%R	%R	%Er	%R	%R	%Er
					Obs.	Pred.		Obs.	Pred.	
01	3 (−)	120 (+)	10 (−)	100 (+)	80.73	78.37	3.01	62.84	61.99	1.37
02	6 (0)	75 (0)	50 (0)	60 (0)	75.34	73.84	2.03	67.48	68.18	1.03
03	6 (0)	75 (0)	50 (0)	60 (0)	72.34	73.84	2.03	69.69	68.18	2.21
04	9 (+)	30 (−)	10 (−)	100 (+)	65.51	65.82	0.47	56.09	54.78	2.39
05	9 (+)	30 (−)	90 (+)	20 (−)	69.79	69.02	1.12	75.95	75.10	1.13
06	3 (−)	120 (+)	90 (+)	20 (−)	82.47	81.08	1.71	83.63	82.32	1.59
07	6 (0)	75 (0)	50 (0)	60 (0)	69.35	73.84	6.08	69.37	68.18	1.75
08	6 (0)	75 (0)	50 (0)	60 (0)	79.26	73.84	7.34	67.89	68.18	0.43
09	6 (0)	75 (0)	50 (0)	60 (0)	75.57	73.84	2.34	70.06	68.18	2.76
10	9 (+)	120 (+)	90 (+)	100 (+)	90.35	92.36	2.18	81.40	79.34	2.60
11	6 (0)	75 (0)	50 (0)	60 (0)	76.13	73.84	3.10	68.77	68.18	0.87
12	3 (−)	30 (−)	10 (−)	20 (−)	47.72	48.74	2.09	59.82	57.76	3.57
13	6 (0)	75 (0)	50 (0)	60 (0)	69.36	73.84	6.07	69.01	68.18	1.22
14	3 (−)	30 (−)	90 (+)	100 (+)	82.22	81.98	0.29	60.91	59.38	2.58
15	6 (0)	75 (0)	50 (0)	60 (0)	72.68	73.84	1.57	68.30	68.18	0.18
16	9 (+)	120 (+)	10 (−)	20 (−)	62.90	64.69	2.77	79.24	77.72	1.96

Obs.: observed responses; Pred.: predicted responses; %Er = ǀ (Experimental reading – Predicted reading)/Predicted reading ǀ⨯ 100.

### 2.7 Investigations of equilibrium and sorption kinetics

To explore the adsorption equilibrium, RIFM and TIGC stock solutions of 500-ppm were first prepared and then diluted to the range of 5–400 ppm. The pH of all dilutions was adjusted to a value of 3.0 ± 0.2 using 0.1 M aqueous solution of HCl. A mass of 0.100 ± 0.005 g of Ce–Py–MSK was inserted in each of the above solutions. The adsorbent-adsorbate mixtures were left to equilibrize at 150 rpm and for 48 h. Samples were filtered, and the filtrate absorbance was recorded at *λ*_max_ of 255 (RIFM) and 347 nm (TIGC).

Exploration of the sorption kinetics was executed by combining 200 mL aliquots of RIFM and TIGC solutions (200 ppm, pH 3.0 ± 0.2) with an amount of ∼1 g of the adsorbent with stirring. Aliquots of 10 mL were withdrawn at different reaction times over an interval of 2 h. Withdrawn aliquots were filtered using syringe filters, and the filtrate absorbance was taken at the *λ*_max_ values indicated under equilibrium measurements.

### 2.8 Desorption and regeneration studies

The desorption and the probability of Ce–Py–MSK regeneration were explored. In this context, 2.0 g of Ce–Py–MSK was allowed to equilibrate for 2 h with 220 mL of RIFM solution (30 ppm). The Ce–Py–MSK–RIFM mixture was filtered and washed with DW to remove any free RIFM. The clean mixture was dried at 70 °C for 48 h. Six different eluents: H_2_O, ethanol, 0.1 M Na_2_CO_3_, 0.1 M H_2_SO_4_, 0.1 M HNO_3_ and 0.1 M HCl were used to elute the loaded Ce–Py–MSK. Desorption investigations were executed by blending 0.1 g of Ce–Py–MSK–RIFM with 10 mL of each of the prepared eluents at 150 rpm for 30 min. Samples were then filtered, and the filtrate absorbance was recorded. The same procedures for desorption studies were reiterated thrice for each eluent and the average of the desorbed amount and the standard deviations were determined and symbolized with error bars. According to the desorption findings, the best eluent will be decided. For the regeneration procedure, a mass of 0.2 g of Ce–Py–MSK was allowed to equilibrate with 25 mL of 30 ppm RIFM for 1 h and pH 9.0 ± 0.2. The resulting suspension was filtered, and the filtrate absorbance was recorded at 255 nm. This process was renewed for 5 cycles, and the %R was calculated in each cycle.

### 2.9 Selectivity of Ce–Py–MSK

The selectivity of Ce–Py–MSK nanocomposite for RIFM and TIGC was assessed by comparing the removal efficiency of Ce–Py–MSK towards both RIFM and TIGC with the removal efficiency for six other drugs (amantadine, ibuprofen, acetaminophen, sofosbuvir, acyclovir, ivermectin), and three organic dyes (rose bengal, fuchsine and methylene blue) [56]. In this regard, aliquots of 13 mL from each contaminant with concentration of 50 ppm was mixed with 0.100 ± 0.005 g of Ce–Py–MSK. The pH of each contaminant solution was adjusted to 9.0 ± 0.2 using 0.1 M aqueous NaOH and the mixture was stirred at 150 rpm for 2 h. Samples were filtered, and the filtrate absorbance was recorded at *λ*_max_ of each adsorbate solution.

## 3 Results and discussion

### 3.1 Preliminary investigation of the adsorption performance of Py–MSK and Ce–Py–MSK

The performance of the two adsorbents (Py–MSK and Ce–Py–MSK) towards the removal of RIFM and TIGC was initially assessed. Two parameters (%R and *q*_*e*_, mg/g) were applied to ascertain the performance of both adsorbents under the preliminary experimental conditions – non-optimized. The data shown in Table 4 reveals that the nano-ceria laden adsorbent (Ce–Py–MSK) has higher adsorption efficiency for both RIFM (76.34%) and TIGC (65.08%) compared to the pristine biochar (Py–MSK). Consequently, Ce–Py–MSK was used throughout this work for the remediation of water contaminated with either RIFM or TIGC.

**Table 4:** Assessment of the performance of Py–MSK and Ce–Py–MSK for the removal of RIFM and TIGC. %R and q_e_ (mg/g) were computed operating Eqs. (1), (2). Experiments were performed under the non-optimized conditions: pH = 7, AD = 80 mg, [Drug] = 60 ppm, and CT = 50 min.

Adsorbent	%R_RIFM_	%R_TIGC_	*q*_*e*__RIFM_ (mg/g)	*q*_*e*__TIGC_ (mg/g)
Py–MSK	64.91	31.66	6.32	3.09
Ce–Py–MSK	76.34	65.08	7.44	6.34

### 3.2 Physicochemical characterization

#### 3.2.1 Thermogravimetric analysis (TGA)

**Fig. 1:**
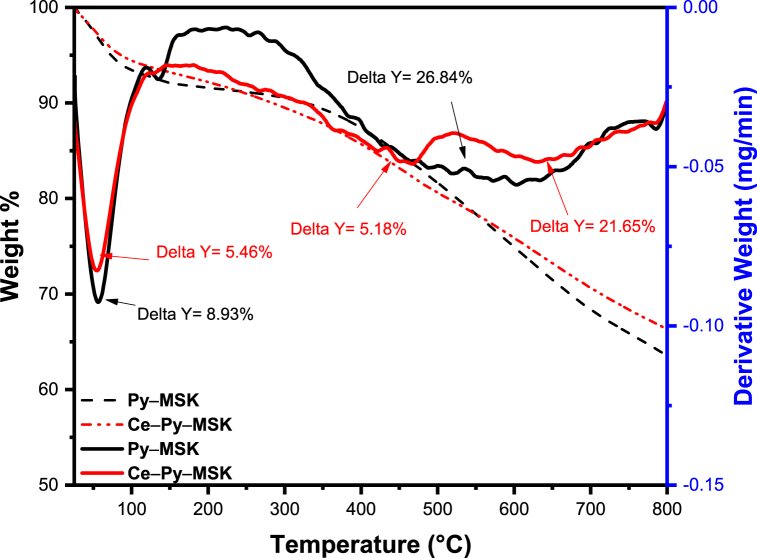
TGA/*d*TA spectra of Py–MSK and Ce–Py–MSK.

Findings of the TGA/*d*TA are revealed in Fig. 1. The acquired results show that the weight loss at the range of 50–200 °C was 8.93% and 5.46% for the Py–MSK and the Ce–Py–MSK, respectively. This loss might be correlated to the free water vaporization. The weight loss at 350–800 °C was almost identical for both adsorbents, totaling 26.84% for Py–MSK and 26.83% for Ce–Py–MSK. This weight loss could be ascribed to the organic matter loss and carbonization of the polymeric content in both samples.

#### 3.2.2 FT-IR characterization

**Fig. 2:**
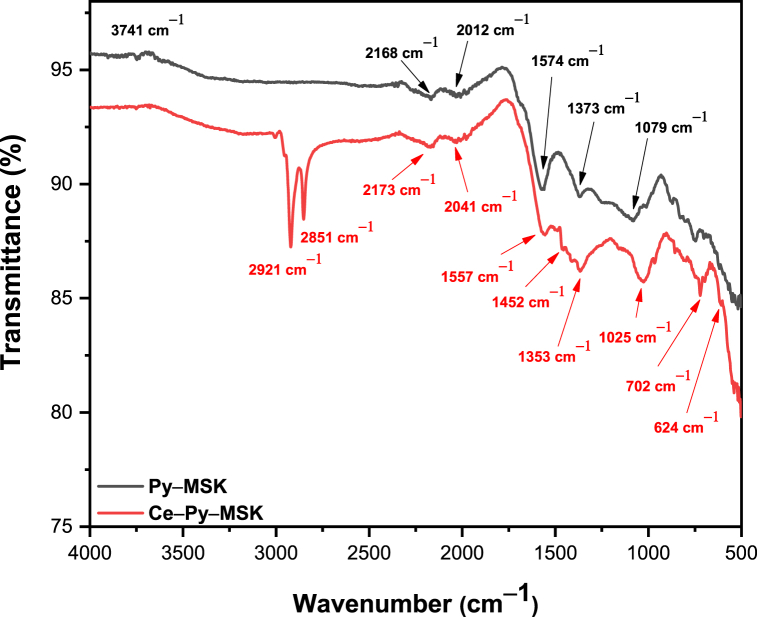
FT-IR spectra of Py–MSK and Ce–Py–MSK.

Fig. 2 shows the occurrence of similar moieties on the surfaces of both adsorbents with slight shifts in peak position. The two weak peaks at 2168, and 2012 cm^−1^ in case of Py–MSK are examples and may be attributed to the presence of primary aliphatic amine. Similar peaks exist on the surface of Ce–impregnated biochar with a small shift (at 2173 and 2041 cm^−1^). The peak at 1574 cm^−1^ in case of Py–MSK corresponds to the ring vibration in the aromatic skeleton, while the band at 1373 cm^−1^ is credited to the oxygen-comprising moieties, e.g., C=O and –C–O bond stretch of ketones and the carboxylic acids. Besides, the peak at 1079 cm^−1^ is ascribed to C–O vibration in alcohols [55,57,58]. In the same rehearsal, several new peaks appear on the surface of Ce–Py–MSK, which could be related to the synthetic processes, including the two bands at 2921 and 2851 cm^−1^, which could be attributed to the alcoholic O–H or amines N–H stretching. Existence of these two peaks could have resulted from the addition of oleylamine, and alcohol during the preparation of nano-ceria. Peak at 1025 cm^−1^ is due to the Ce–O–C and the peak at 702 cm^−1^ could be ascribed to the Ce–O vibrational mode, confirming the existence of nano-ceria on the surface of the decorated biochar [[59], [60], [61], [62]].

#### 3.2.3 Raman analysis

**Fig. 3:**
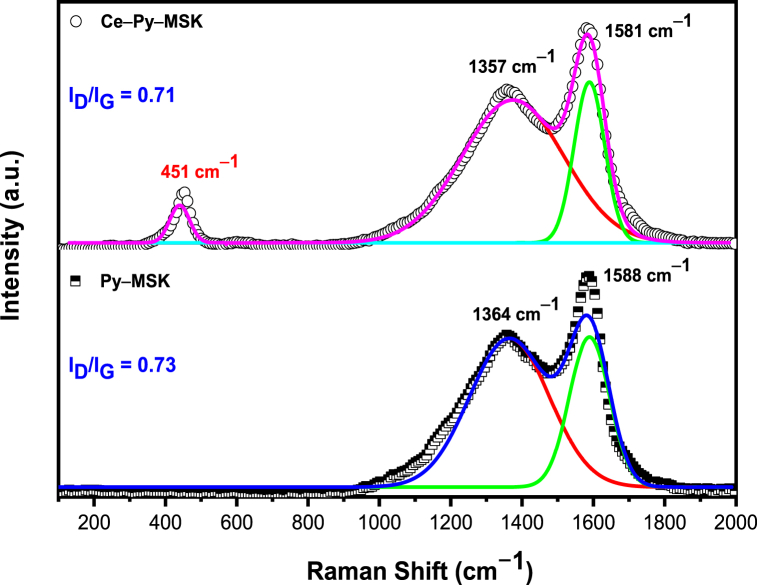
Raman spectra of Py–MSK and Ce–Py–MSK.

Fig. 3 shows the Raman spectra of Py–MSK and Ce–Py–MSK. The spectra reveal two significant bands at Raman shifts of 1357 and 1581 cm^−1^ that could be attributed to the D– and G–bands, correspondingly. The D–band at 1359 cm^−1^ is correlated to the defects in the graphitic structure [63]. However, the G–band at 1585 cm^−1^ is associated with the C=C stretching of graphitic structure, and it helps determining the level of graphitization. Furthermore, the obtained data show that the *I*_*D*_*/I*_*G*_ ratio for Py–MSK is 0.73, compared to 0.71 for Ce–Py–MSK, confirming the existence of nano-ceria on the surface, which in turn helps decreasing the degree of defects on the prepared adsorbent. Consequently, this could affect the adsorption competency of the prepared sample. Moreover, the Raman spectrum of the Ce–Py–MSK reveals a strong peak at 451 cm^−1^ which could be linked to the occurrence of cubic ceria nanoparticles. The peak broadness in case of ceria–impregnated adsorbent points to the small particle size [64,65]. These findings confirm the occurrence of cubic nano-ceria on the surface of the Py–MSK.

#### 3.2.4 X-ray diffraction analysis (XRD)

**Fig. 4:**
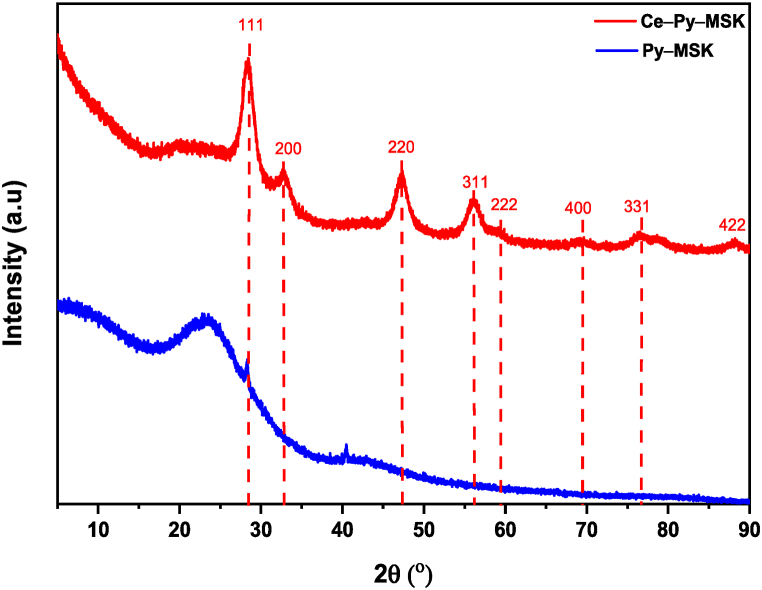
XRD analysis of the as-prepared samples: Py–MSK and Ce–Py–MSK.

X-ray diffraction analysis is an important analytical approach for figuring out the crystalline phase of any powder material. X-ray diffraction analysis was used to investigate the samples in order to confirm the crystallographic structure of ceria nanoparticles impregnated onto Py–MSK. Fig. 4 depicts the XRD patterns of Py–MSK and Ce–Py–MSK. For the Py–MSK sample, the XRD patterns reveal a broad peak in the 2θ 17°–30°, predicting the presence of an amorphous state of Py–MSK. This pattern was also observed in the XRD diffractogram of Ce–Py–MSK, demonstrating the existence of a carbon layer with CeO_2_ nanoparticles [66]. Furthermore, the XRD diffractogram of Ce–Py–MSK reveals three strong peaks confirming the presence of cubic CeO_2_ (ICDD: 98-002-8709) at 2θ 28.67°, 47.69°, and 56.60° corresponding to the (111), (220) and (311) planes of CeO_2_ nanoparticles, respectively [67,68]. The XRD findings further ensure the existence of cubic CeO_2_ nanoparticles on Ce–Py–MSK.

#### 3.2.5 Surface area and pore structure analysis

**Fig. 5:**
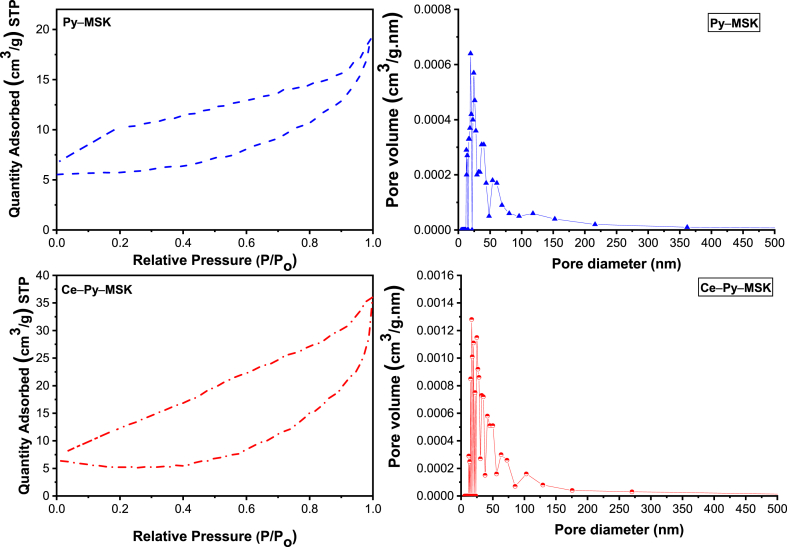
BET analysis and the adsorption pore volume for Py–MSK (blue) and Ce–Py–MSK (red). (For interpretation of the references to color in this figure legend, the reader is referred to the Web version of this article.)

Fig. 5 demonstrates the outcomes of the BET analysis of the two adsorbents. The attained data indicate that the Langmuir surface area was 24.72 m^2^/g and 33.83 m^2^/g for Py–MSK and Ce–Py–MSK, correspondingly, an issue that corroborates that the surface area has risen because of the manifestation of the nano-ceria. This in turn has been reflected on the adsorption efficiency towards RIFM and TIGC. Furthermore, the findings show the occurrence of 2 types of pores in both adsorbents: one type in the range 2–50 nm (mesopores) and second type has pore size >50 nm (macropores). Pore volume has raised from 0.030 cm^3^/g in Py–MSK to 0.056 cm^3^/g in Ce–Py–MSK, and the mesopores–to–macropores ratio in Py–MSK and Ce–Py–MSK was high. The adsorption isotherm for the two adsorbents, which is usually correlated with the adsorption in microporous and mesoporous materials, was of type IV. These findings indicate that capillary condensation might have occurred as part of the adsorption process. Gases condense in the small capillary pores of the material at pressures less than the gas equilibrium pressure. It also implies the formation of a monolayer followed by the formation of multilayers at lower pressures. This type of isotherm is characteristic of mesoporous materials. Furthermore, the hysteresis loop of both samples was of the H3 form, which is usually observed in materials with a complex pore structure [69].

#### 3.2.6 SEM, EDX and TEM characterization

**Fig. 6:**
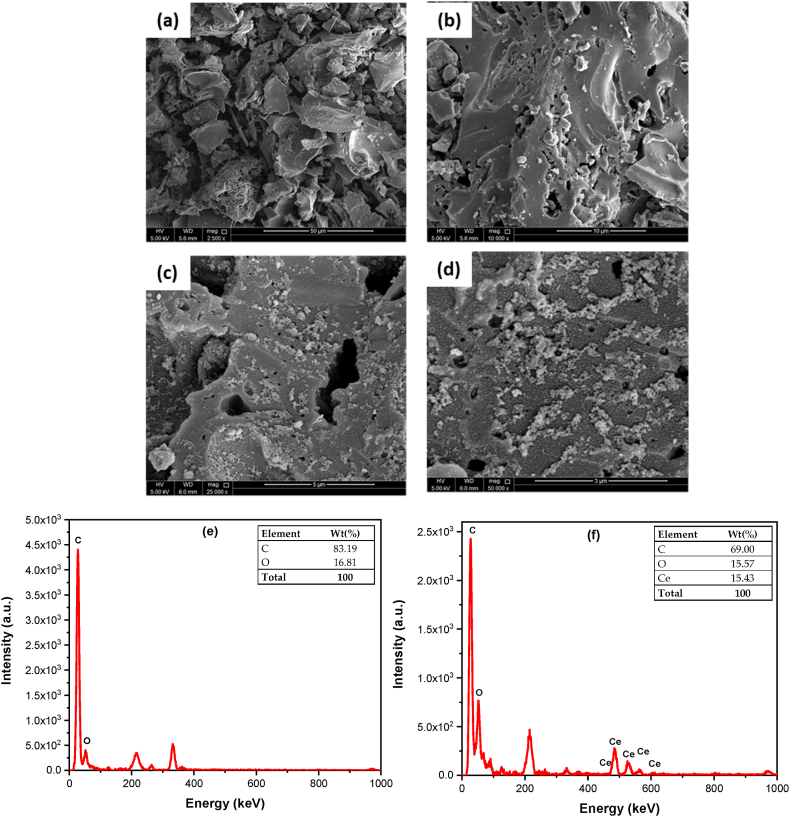
SEM micrographs of **(a, b)** Py–MSK (at 2500 × and 10 000× magnifications), **(c, d)** Ce–Py–MSK (at 25 000 × and 50 000× magnifications), **(e, f)** EDX analyses of Py–MSK and Ce–Py–MSK, correspondingly.

The composition and morphology of the prepared sorbents was evaluated using SEM, EDX, and TEM techniques. The existence of pores with different sizes was confirmed by the SEM micrographs of the Py–MSK, Fig. 6a and b, supporting the evidence obtained from the BET study. SEM micrographs of Ce–Py–MSK shown in Fig. 6c and d demonstrate the existence of fine CeO_2_ nanoparticles, proving the deposition of nano-ceria on the biochar surface.

Furthermore, the findings of SEM micrographs were confirmed using EDX analysis, Fig. 6e and f. Presence of ceria in Ce–Py–MSK with a concentration of 15.43% compared to *null* in case of Py–MSK sample, confirms the loading of CeO_2_ on the surface. Moreover, the data revealed in Fig. 6e and f Show that carbon concentration decreased from 83.19% in Py–MSK to 69.00% in case of Ce–Py–MSK, most probably because nano-ceria have apparently replaced part of the carbon.

**Fig. 7:**
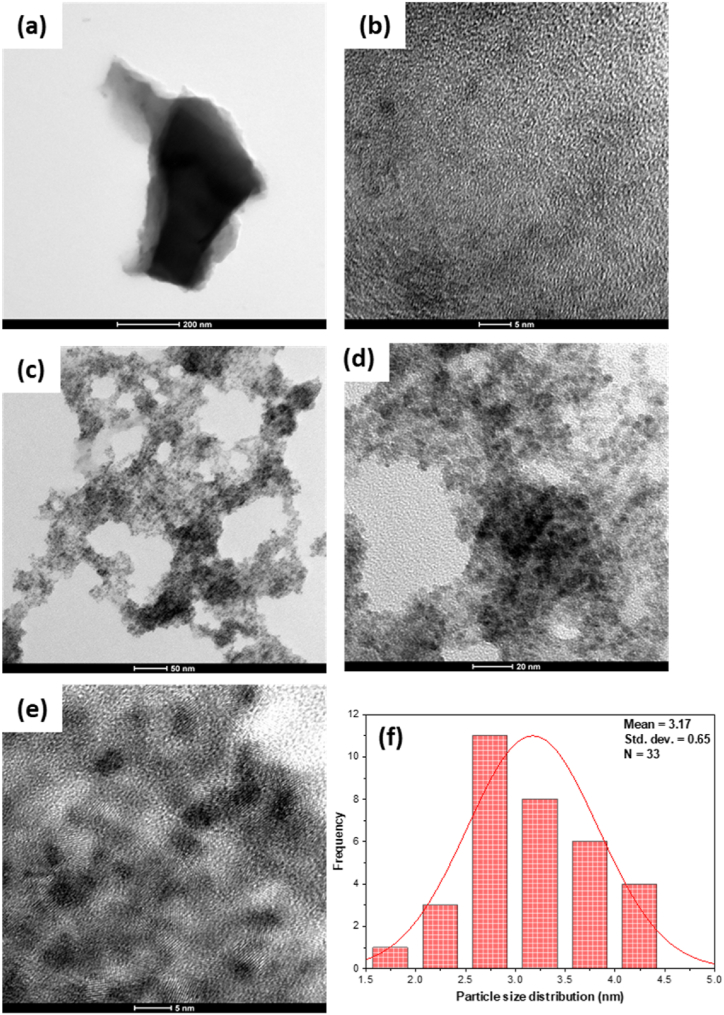
TEM graphs of the Py–MSK at **(a)** 200 nm, **(b)** 5 nm, Ce–Py–MSK at **(c)** 50 nm, **(d)** 20 nm and **(e)** 5 nm, and **(f)** particle size distribution (PSD) for Ce–Py–MSK.

TEM technique was used to investigate the microstructure of Py–MSK and Ce–Py–MSK. Fig. 7a and b demonstrate that Py–MSK surface is plain and free of particles. The micrographs of the Ce–Py–MSK sample indicate the presence of cubic CeO_2_ (Fig. 7c, d, and e). Particle size distribution (Fig. 7f) showed that the average particle size of CeO_2_ nanoparticles was 3.17 ± 0.65 nm. The fact that the SD of ceria nanoparticles was very small (0.65) indicates the uniformity of size. These data confirm the obtained findings from the previous characterization techniques, including SEM, EDX, and BET analyses.

### 3.3 Design analysis

The purpose of this investigation is to compose a cost-effective adsorbent by reusing of locally available waste materials; MSK. Furthermore, the suggested objectives entailed increasing the adsorption effectiveness of the MSK–derived biochar sorbent (ceria–laden) through a green, and lucrative approach. Following the selection of MSK biochar decorated with ceria nanoparticles, an experimental design was built to screen the factors impacting the adsorption process for RIFM and TIGC. A multivariate analysis supported technique (2^*k−p*^ FrFD) was used to construct a model adsorbent, where *k* denotes the number of variables and *p* represents the fraction index [51,52]. The design matrix and the obtained response together with the calculated %error (%Er) weighed against the forecasted values are displayed in Table 3. As could be comprehended, the values of %R (predicted) were near enough to the experimental ones as indicated by the low values of %Er.

#### 3.3.1 Quality charts and analysis of variance (ANOVA)

**Fig. 8:**
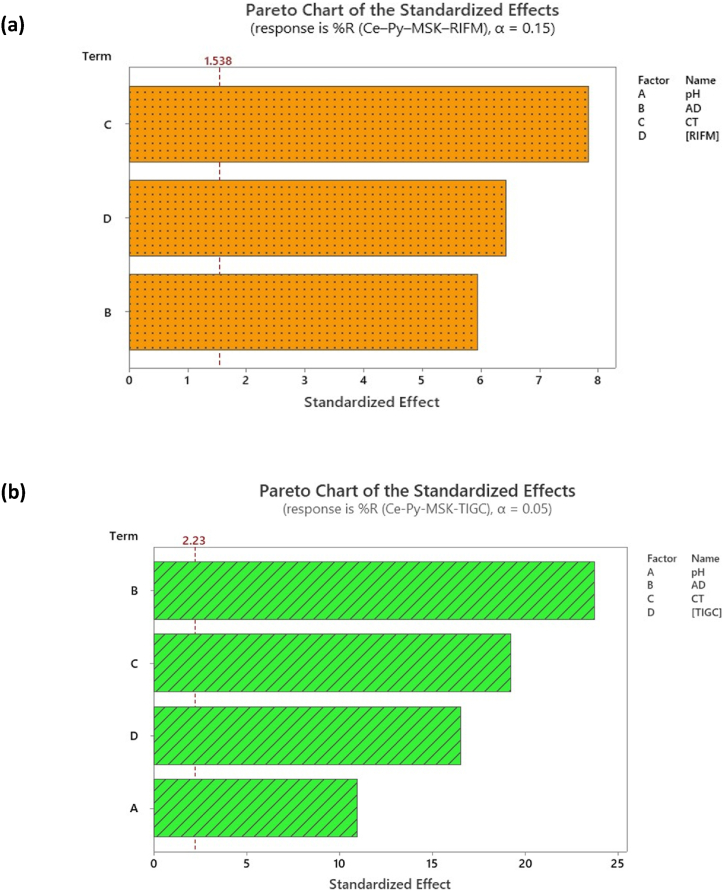
Pareto chart of standardized effects using Ce–Py–MSK as adsorbent in case of **(a)** RIFM, and **(b)** TIGC. Data were obtained following response transformation.

Pareto chart of standardized variables, Fig. 8 serves to emphasize the statistical importance of the analyzed variables. From the chart, it can be concluded that in the case of RIFM (Fig. 8a), the linear effect of CT (C) is the most statistically significant variable, while pH (A) is not statistically significant. In the case of TIGC (Fig. 8b), howbeit, AD (B) was the most substantial effect. The same conclusions were corroborated by charting the normal and half-normal plots (figures are not presented) in addition to the ANOVA testing.

#### 3.3.2 Contour and surface plots

**Fig. 9:**
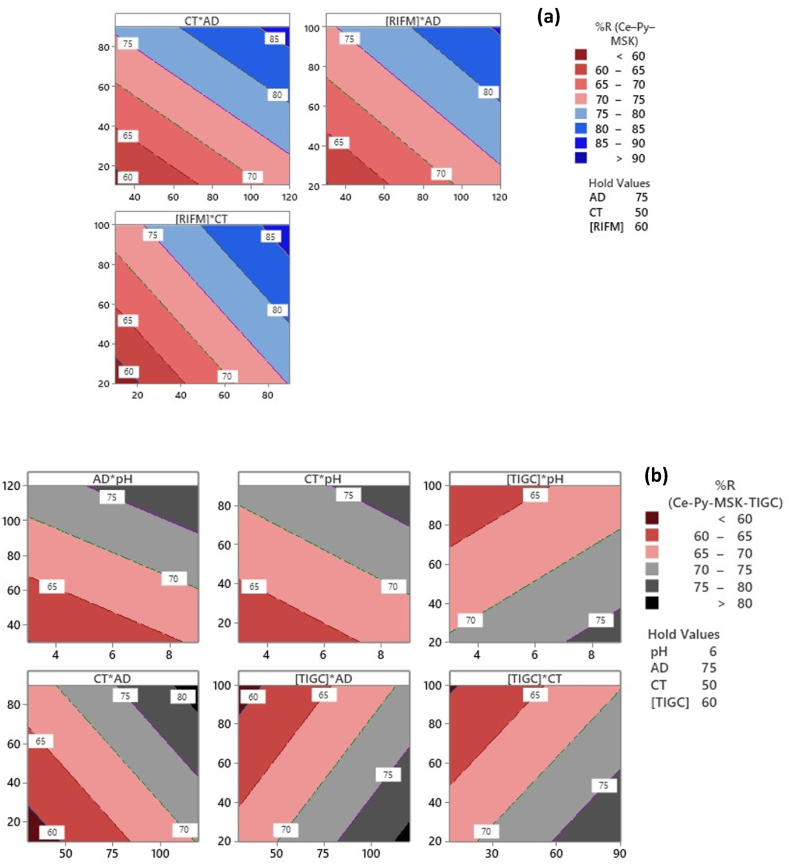
Response contour plots for **(a)** RIFM, and **(b)** TIGC.

Fig. 9 shows the contour plots (two-dimensional plots) for both drugs. As shown, the dark blue color signifies a high %R for RIFM (Fig. 9a) compared to dark grey color in the case of TIGC (Fig. 9b). For example, in Fig. 9a – upper left panel shows the relation of two independent variables CT⨯AD to the response variable. Using of a CT of ≥80 min, and AD of ∼ <110 mg/mL could achieve a %R of > 85%. Surface plots – Figures are not shown, are commonly used to express the same a relation, however, in a 3D format.

#### 3.3.3 Regression equations and response optimization

Eqs. (3), (4) represent the design output in case of RIFM and TIGC, respectively. Regression equations were obtained following the application of Box-Cox response transformation with a transformation factor of 2 in case of RIFM (accompanied with stepwise analysis) and no transformation in case of TIGC [70]:

(3) %R^2^_(Ce–Py–MSK–RIFM)_ = 984 + 20.11 AD + 29.85 C T + 24.46 [RIFM]

(4) %R_(Ce–Py–MSK–TIGC)_ = 52.446 + 1.0619 pH + 0.15095 AD + 0.13719 C T − 0.11690 [TIGC] − 1.161 C t Pt

Summary of the developed models are displayed in Table 5. As could be deduced from the displayed results, the high value of R^2^ indicates model linearity. Model prediction capability could be observed from the high value of R^2^ (predicted). This finding can be also established from Table 3. The discrepancies between R^2^ (adjusted) and R^2^ (predicted) were < 10% implying that there is no model over-fitting.

**Table 5:** FrFD summary and optimum conditions.

Parameter	%R_RIFM_	%R_TIGC_
Coefficient of determination, R^2^	91.99	99.34
R^2^ (adjusted)	89.99	98.90
R^2^ (predicted)	87.95	98.46
Optimum conditions	AD = 120 mg/13 mL, CT = 90 min, [RIFM] = 100 ppm	pH = 9.0 ± 0.2, AD = 120 mg/13 mL, CT = 90 min, [TIGC] = 20 ppm
Desirability function (*d*)	1.0000, %R_RIFM_ = 92.36%	1.0000, %R_TIGC_ = 90.13%

In the same itinerary, model optimization was achieved using the individual desirability function (*d*). As shown in Table 5, the value of *d* was 1.0000 for both drugs inferring that the listed blend of variables are the optimum conditions that could be used to achieve the maximum %R. While pH has no influence in case of RIFM, a value of pH = 9.0 ± 0.2 was optimum in case of TIGC. On the other hand, the obtained data shown in Table 6 reveals the reproducibility of the response (%R) obtained under the optimum conditions and confirm the precision of the factorial design findings, as inferred by the small values of the obtained standard deviation (SD).

**Table 6:** Repeated trials at the optimum conditions for the adsorption RIFM and TIGC onto Ce–Py–MSK.

Trial #	%R_RIFM_	SD	%R_TIGC_	SD
1	92.41	0.42	89.07	0.79
2	92.92		88.18	
3	93.28		88.20	
4	92.21		87.81	
5	92.64		89.75	

### 3.4 Equilibrium and kinetics investigations

#### 3.4.1 Equilibrium isotherms

Investigation of the adsorption equilibrium was performed to assess the nature of adsorbate-adsorbent interactions, and to measure the adsorbate aggregation onto the adsorbent surface. In this regard, four models: Langmuir, Freundlich, Temkin and Dubinin-Radushkevich (D-R) isotherms were exploited to study the adsorption of RIFM and TIGC onto Ce–Py–MSK [11,31,71,72].

**Fig. 10:**
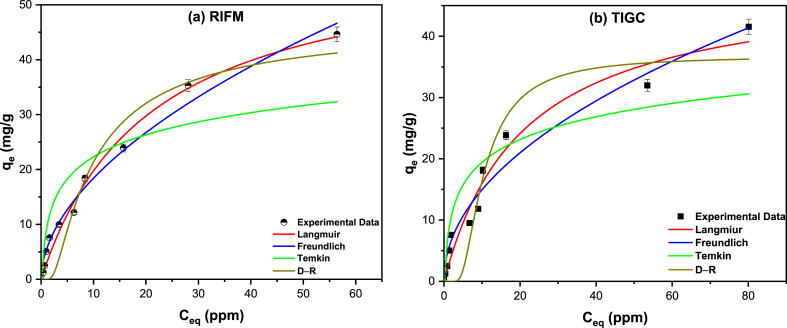
Adsorption isotherms of RIFM **(a)** and TIGC **(b)** onto Ce–Py–MSK. The measurements were performed in triplicate, and the results were presented as the mean value ± SD.

Starting with Langmuir equilibrium isotherm, the following three hypotheses might be used to explain the isotherm data: 1. The heat of adsorption of all sorption sites is equal and is not dependent on the number of sites; 2. There is no interaction with the adsorbate; and 3. The adsorption process is localized. Eq. (5) can be used to portray Langmuir isotherm for the adsorption of RIFM (Fig. 10a) and TIGC (Fig. 10b) onto Ce–Py–MSK.

(5) $$q_{e}=\frac{q_{m}\;K_{L}\;C_{e}}{1+K_{L}\;C_{e}}$$

In Eq. (5), the maximum adsorption capacity is denoted as *q*_*m*_, and the Langmuir constant is given as *K*_*L*_. The dimensionless formula (Eq. 6) can be used to express the Langmuir isotherm model:

(6) $$R_{L}=\frac{1}{1+K_{L}\;C_{0}}$$

Where RL is the separation factor and C0 (ppm) is the initial concentration of both RIFM and TIGC. Adsorption favorability of adsorbate onto Ce–Py–MSK was determined using the RL value; hence, if RL equals 1, the adsorption is unfavorable, if RL = 1, the adsorption is linear, and if the value is between 0 and 1, then the adsorption process is favorable. If RL = 0, then the adsorption process is irreversible. The obtained data shows that the RL value for both drugs was <1, meaning that their adsorption onto Ce–Py–MSK occurs spontaneously. The maximum adsorption capacity (*q*_*m*_) for both drugs was calculated to be 102.25 mg/g for RIFM and 49.28 mg/g for TIGC, indicating that the examined adsorbent (Ce–Py–MSK) has a better removal performance towards RIFM than TIGC and confirms the FrFD findings.

The Freundlich isotherm, Eq. (7), can be applied to represent the energy of any heterogeneous surface:

(7) $$q_{e}=K_{F}C_{e}^{\frac{1}{n}}$$

In this equation, *K*_*F*_ and 1/*n* are the Freundlich constants. The R^2^ values were 0.9855 and 0.9675 for both RIFM and TIGC, respectively (Fig. 10a and b, Table 7). R^2^ values were greater than the other equilibrium isotherms; hence, the Freundlich isotherm could be utilized to interpret both the adsorption of RIFM and TIGC onto Ce–Py–MSK. Furthermore, the calculated data for RIFM revealed that *1/n* = 0.54 and *n* = 1.85 compared to *1/n* = 0.49 and *n* = 2.04, for TIGC. As a result, the RIFM and TIGC adsorption potentials (A = *n*RT) were 6.08 and 6.70, correspondingly. Therefore, any RIFM or TIGC molecule with a potential energy lower than these values may be adsorbed onto Ce–Py–MSK surface, and the adsorption process is favorable and irreversible.

**Table 7:** Calculated parameters of the equilibrium isotherms, for the adsorption of both drugs onto Ce–Py–MSK.

Model	Nonlinear Equations	Parameters	Value	
			RIFM	TIGC
Langmuir	$q_{e}=\frac{q_{m}\;K_{L}\;C_{e}}{1+K_{L}\;C_{e}}$	$q_{m}$ (mg/g)	102.25	49.28
		$K_{L}$ (L. mole^−1^)	0.048	0.047
		R^2^	0.9768	0.9667
Freundlich	$q_{e}=K_{F}C_{e}^{\frac{1}{n}}$	1/n	0.54	0.49
		$K_{F}$ (mole/g) (L/mole)^1/n^	5.35	4.84
		R^2^	0.9855	0.9675
Temkin	$q_{e}=\frac{RT}{b_{T}}\;\ln\left(A_{T}\;C_{e}\right)$	$b_{T}$ (J/mole)	426.1	461.5
		$A_{T}$ (L/mole)	4.63	3.73
		R^2^	0.7583	0.7866
D-R	$q_{e}=q_{s}$. Exp (-β. $\varepsilon^{2}$)	β	6.15 × 10^−8^	2.91 × 10^−8^
	$\varepsilon =RT\left(1+\frac{1}{C_{e}}\right)$	E (kJ/mole)	2.85	4.14
		$q_{m}$ (mg/g)	40.59	36.76
	E=12β	R^2^	0.9229	0.9154

Q_m_: maximum adsorption capacity, K_L_: Langmuir constant, K_F_, 1/n: Freundlich constants, A_T_, b_T_: Temkin constants, R: universal gas constant, T: temperature, q_s_: saturation capacity, β: activity coefficient, and ε: calculated Polanyi potential.

Temkin model (Fig. 10, Table 7) helps to give guidance on the interaction of the two drugs with Ce–Py–MSK. As a result of the adsorbent-adsorbate interactions, the heat of adsorption of the adsorbed molecule in the layer might drop linearly. Based on the findings, the sorption energy of RIFM and TIGC is 426.1 and 461.5 J/mol, respectively, suggesting preferential adsorption of both RIFM and TIGC over the investigated adsorbent and validating the data from Langmuir isotherms.

The D-R isotherm data for both drugs at ambient temperature are shown in Fig. 10, and Table 7. Calculated data revealed that the sorption energy for RIFM equals 2.85 kJ/mol comapred to 4.14 kJ/mol for TIGC, insinuating that the adsorption of both drugs onto Ce–Py–MSK is physisorption, and is mostly controlled by the surface area of the biochar as well as the existence of porous structure on its surface. However, the occuerence of chemisorption cannot be ignored because of the presence of several functional groups on the surface of Ce–Py–MSK as confrimed by the FT-IR.

#### 3.4.2 Kinetic studies

**Fig. 11:**
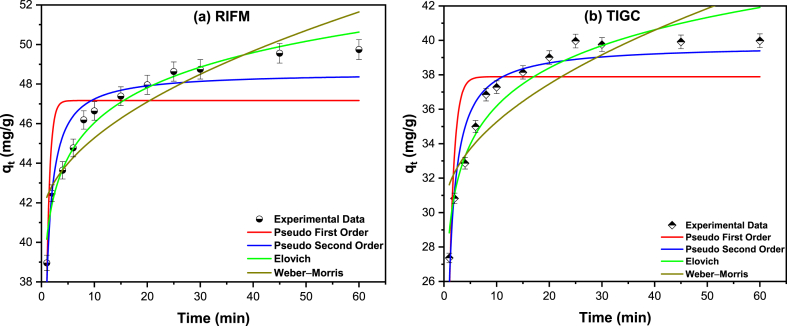
Kinetic studies for the adsorption of **a)** RIFM and **b)** TIGC onto Ce–Py–MSK. The measurements were performed in triplicate, and the results were presented as the mean value ± SD.

Kinetic studies were performed using four models, namely pseudo first and second order (PFO and PSO), Elovich, and Weber-Morris models [[73], [74], [75]]. The relationship between *q*_*t*_ (mg/g) versus time (min) was plotted for sorption of the two drugs (Fig. 11 a and b, Table 8) onto Ce–Py–MSK. The acquired data for the PFO and PSO models reveal that the R^2^ value is greater for the PSO model compared to the PFO, 0.8954 for RIFM and 0.9343 for TIGC. Therefore, the rate of reaction between the two drugs and Ce–Py–MSK relies mostly on both the drug and the adsorbent. The overall interaction may be described as follows, Eq. (8):

(8) $$RIFM\;\&\;TIGC+Ce–Py–MSK\;\left(\overset{k}{\to }\right)\;\left\{RIFM–Ce-Py–MSK\right\}or\;\left\{TIGC–Ce–Py–MSK\right\}$$

**Table 8:** Kinetics studies for the adsorption of the two drugs onto Ce–Py–MSK.

Isotherm	Parameter	Value	
		RIFM	TIGC
Pseudo first order (PFO)	K_1_ (min^−1^)	1.609	1.062
$\frac{dq_{t}}{dt}$ = *k*_*1*_*(q*_*e−*_*q*_*t*_*)*	*q*_*e*_ (mg/g)	47.16	37.89
	R^2^	0.6038	0.6782
Pseudo second order (PSO)	K_2_*(*g.mg^−1^.min^−1^*)*	0.072	0.046
$\frac{dq_{t}}{dt}$ = *k*_*2*_*(q*_*e−*_*q*_*t*_*)*^*2*^	*q*_*e*_ (mg/g)	48.59	39.75
	R^2^	0.8954	0.9343
Elovich model	α	1.69 × 10^7^	2.65 × 10^4^
*q*_*t*_ = $\frac{1}{\beta }\times \ln\left(1+\alpha \beta t\right)$	β	0.391	0.312
	R^2^	0.9682	0.9356
Weber-Morris model	K_I_	1.389	1.685
$q_{t}=K_{I}t^{0.5}+C$	C	40.88	29.93
	R^2^	0.8112	0.7383

Q_t_: capacity at t, K_1_: adsorption rate constant, t: adsorption time, K_2_: adsorption rate constant, α, β: Elovich constants, K_I_: intraparticle diffusion rate constant, C: thickness of the boundary layer.

By and large, the diffusion mechanism can be delineated employing Elovich model rather than the PFO and PSO models. The Elovich model could predict the diffusion mechanism using the rate of drug adsorption on the adsorbent surface; where the adsorption rate rapidly increases reaching the maximum level and implying that the available surface area of the adsorbent becomes saturated with the drug molecules. This model also suggests that the strength of the interaction between the drug molecules and the surface can be quantified by the Elovich constant, which is related to the activation energy of the adsorption process. By fitting experimental data to the Elovich model, it can be observed that both RIFM and TIGC adsorption conforms well with the Elovich model (in addition to the PSO model), and R^2^ value equals 0.9682 and 0.9356 for RIFM and TIGC, respectively. Likewise, a strong initial adsorption for the two drugs, with α-values of 1.69 ⨯ 10^7^ and 2.65 ⨯ 10^4^ mg g^−1^. min^−1^, could be observed for RIFM and TIGC, correspondingly. Finally, the R^2^ value for the Weber-Morris model was too low for both drugs compared to other models (0.8112 for RIFM and 0.7383 for TIGC). Therefore, this model cannot be utilized to describe the adsorption of RIFM or TIGC onto Ce–Py–MSK.

### 3.5 Proposed adsorption mechanism

The following mechanisms could be used to describe the adsorption of RIFM and TIGC onto Ce–Py–MSK: hydrogen-bonding, electrostatic interactions, intra-particle, and surface diffusions [76]. Electrostatic interactions occur when the charged functional groups of the RIFM and TIGC interact with the charged surface of Ce–Py–MSK. These interactions can be influenced by the pH, where the change in pH can alter the surface charge of the both the drug and the adsorbent. Hydrophobic interactions occur when the hydrophobic portions of the drug molecule interact with the hydrophobic surface of Ce–Py–MSK. This mechanism is significant for drugs with long hydrophobic chains, such as TIGC. Finally, chemical bonding can occur between the drugs and Ce–Py–MSK surface through covalent or ionic bonds. This mechanism is particularly important for RIFM, which has functional groups that can participate in chemical reactions with the surface of Ce–Py–MSK. The presence of different functional groups with benzene or other aromatic ring structure of the adsorbate, which could have electron-rich or electron-deficient groups that facilitates the interaction. Additionally, there may be hydrogen-bonding interactions between the nitrogen and oxygen atoms (H-acceptors) in Ce–Py–MSK and the hydrogen of the hydroxyl groups (H-donors) on both RIFM and TIGC. This type of interaction is commonly referred to as hydrogen bonding between dipoles.

**Fig. 12:**
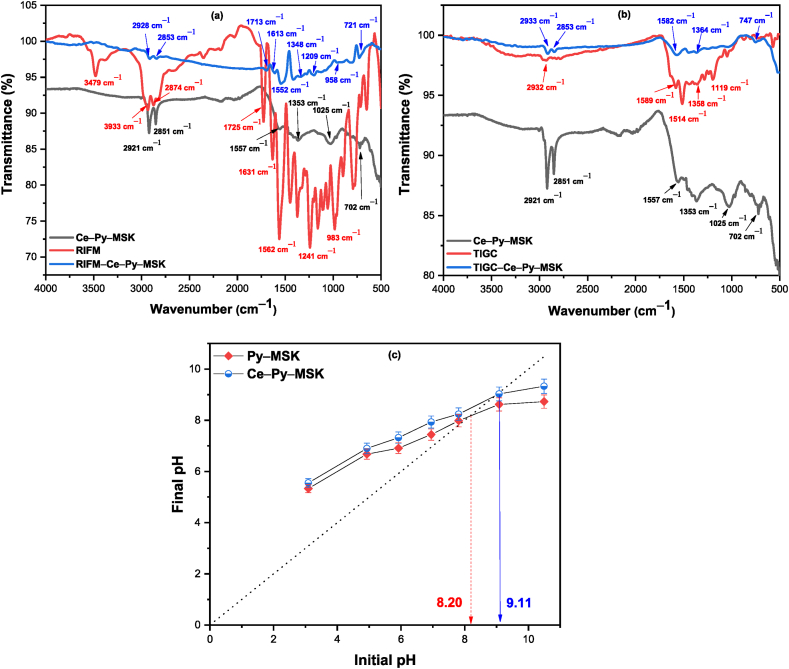
(A) RIFM, Ce–Py–MSK and RIFM@ Ce–Py–MSK after adsorption, **(b)** TIGC, Ce–Py–MSK and TIGC@ Ce–Py–MSK after adsorption, **(c)** pH_PZC_ of Py–MSK and Ce–Py–MSK. The pH_PZC_ measurements were performed in triplicate, and the results were presented as the mean value ± SD.

FT-IR analysis of the two drugs before and after adsorption was used to investigate the proposed sorption mechanisms. Fig. 12a shows the FT-IR spectrum for RIFM and RIFM–Ce–Py–MSK. The acquired data shows the appearance of significant absorption bands of RIFM. The band at 3477 cm^−1^ might be correlated to the N–H or O–H stretching. Furthermore, the peaks at 2934 and 2867 cm^−1^ are related to the C–H and = C–H vibration. The peak at 1725 cm^−1^ could be allocated to C=O group. Also, the C=C stretching might be assigned by a significant absorption band at 1562 cm^−1^ [64,77]. However, the spectrum of Ce–Py–MSK after RIFM adsorption shows the existence of the bulk of the RIFM peaks, indicating that the RIFM was successfully adsorbed onto the biochar surface. Parallel data were obtained after adsorption of TIGC onto Ce–Py–MSK (Fig. 12b). Several TIGC peaks were observed after adsorption with small shifts, e.g., the peak at 1358 cm^−1^ in TIGC was observed at 1364 cm^−1^ demonstrating formation of bond between the function groups present on the surface of the adsorbent and TIGC groups.

The surface charge of both adsorbents was investigated and the obtained data revealed that the pH_PZC_ of Py–MSK was 8.20 ± 0.20 compared to 9.11 ± 0.20 for Ce–Py–MSK, Fig. 12c. Similar results were previously reported to Py–MSK [41]. These elevated figures reflect that the surface of both adsorbents has more basic functional groups. RIFM, on the other hand, has two pKa values: 1.7 (4-hydroxy group) and 7.9 (3-piperazine nitrogen) [78]. As a result, the type of adsorption (chemi- or physisorption) could be decided based on the FrFD results for the optimal pH value as well as the equilibrium data, as represented in the previous sections. In conclusion, the proposed mechanisms of adsorption of RIFM and TIGC on Ce–Py–MSK include electrostatic interactions, hydrophobic interactions, and chemical bonding. These mechanisms can occur simultaneously or separately, and their relative importance depends on the specific drug molecule and the surface properties of Ce–Py–MSK.

### 3.6 Regeneration and desorption studies Ce–Py–MSK

**Fig. 13:**
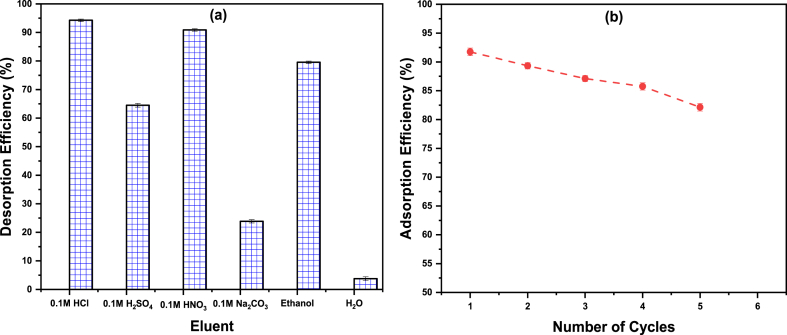
(A) RIFM desorption from Ce–Py–MSK using different eluents, and **(b)** Ce–Py–MSK regeneration for RIFM removal. The measurements were performed in triplicate, and the results were presented as the mean value ± SD.

Adsorbent economic applicability is crucial and is mostly decided by adsorbent restoration. As a result, a desorption process was performed using six eluents, and then five sequential adsorption-desorption cycles. Fig. 13a represents the tested eluents versus the desorption efficiency (%). Depending on the results, the optimum eluent for the loaded Ce–Py–MSK was 0.1 M HCl, and the obtained desorption efficiency is 94.25%. As a result, 0.1 M HCl was chosen as an eluent for RIFM from loaded Ce–Py–MSK.

The findings of cyclic adsorption-desorption tests are displayed in Fig. 13b. The acquired data reveal that the RIFM removal efficiency for the investigated adsorbent decreased slightly, lowering the adsorption efficiency from 92.36% (cycle 1) to 82.13% (cycle 5). These findings show that Ce–Py–MSK could be efficiently regenerated and utilized for more than five cycles with a RIFM removal efficiency of more than 80%.

### 3.7 Adsorption selectivity of Ce–Py–MSK towards RIFM and TIGC

**Fig. 14:**
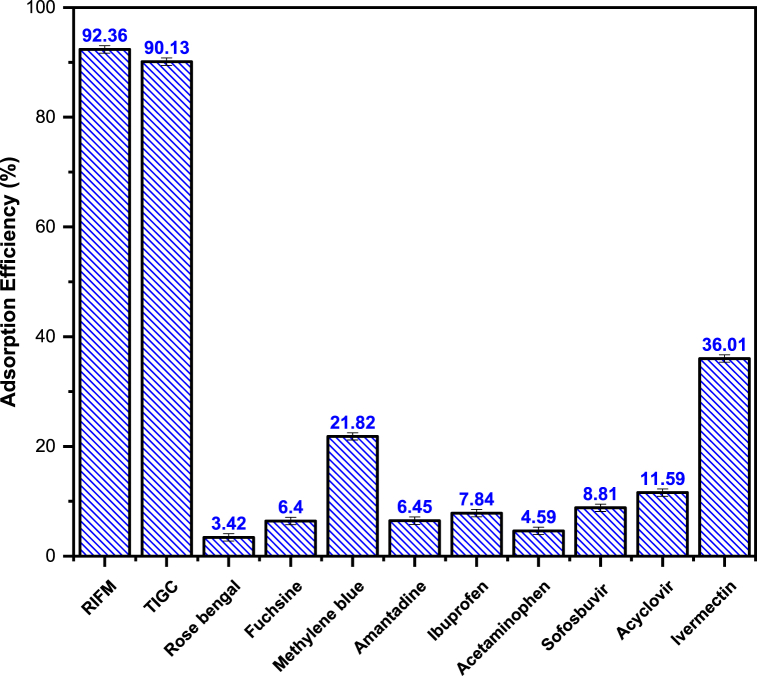
Adsorption selectivity of Ce–Py–MSK towards RIFM and TIGC in comparison with different drugs and organic dyes. The measurements were performed in triplicate, and the results were presented as the mean value ± SD.

To examine the specificity of the sorption process, Ce–Py–MSK was tested for its adsorption efficacy towards RIFM and TIGC and compared to the aqueous solutions of nine drugs and dyes possessing various chemical structures and pharmacological actions. Studied interferents included: amantadine, ibuprofen, acetaminophen, sofosbuvir, acyclovir, ivermectin, rose bengal, basic fuchsine and methylene blue at the same concentration of 50 ppm. Fig. 14 shows that Ce–Py–MSK adsorbs both RIFM and TIGC significantly with adsorption efficiency of 92.36 and 90.13%, respectively. On the other hand, the adsorption efficiency for the other drugs and organic dyes was relatively lower compared to RIFM and TIGC. The highest adsorption efficiency was obtained for ivermectin and methylene blue, with adsorption efficiency of 36.01 and 21.82%, respectively. Besides, the adsorption efficiency of the other adsorbate solutions was less than 11%, revealing a low preference for Ce–Py–MSK towards these interferents compared to RIFM and TIGC.

## 4 Conclusions

Green adsorbents obtained via recycling of agro-wastes have garnered a lot of attention recently. On one hand, the unique properties of waste-derived materials support their application in many environmental remediation rehearsals. From the other hand, valorization of agro-waste relieves the burden on the environment in case waste materials have not been processed. This study aimed at the removal of two antibiotics: RIFM and TIGC, from synthetic wastewater using the biochar of MSK, both in the pristine format (Py–MSK) and following the decoration with nano-ceria (Ce–Py–MSK). To optimize the process variables, a multivariate technique – fractional factorial design (2^*k−p*^ FrFD) was utilized to control the process variables. The objective was set to attain the greatest depollution of the two antibiotics with the least probable depletion of resources and chemicals. Based on the design output, CT is the most statistically significant variable for RIFM, compared to AD in case of TIGC. Obtained data show that Ce–Py–MSK showed a higher adsorption capacity towards both RIFM (92.36%) and TIGC (90.13%), a property that could be attributed to its higher porosity thanks to the nano-ceria on the adsorbent surface. BET analysis confirmed the higher surface area and porosity of Ce–Py–MSK compared to the pristine candidate. Existence of nano-ceria was further confirmed by the Raman, SEM, EDX, and XRD analyses. TEM micrographs showed the presence of nano-ceria as fine and uniform nanoparticles with an average particle size of 3.17 ± 0.65 nm. Furthermore, following the adsorption of RIFM and TIGC, FT-IR analysis indicated a slight shift in the location of various functional groups, demonstrating the presence of both drugs on the adsorbent surface. Nonlinear fittings of the equilibrium data showed that the adsorption of both drugs onto Ce–Py–MSK was favorable, with a qm of 102.25 and 49.28 mg/g for RIFM and TIGC, correspondingly. The D–R equilibrium isotherm showed that the adsorption of both drugs onto Ce–Py–MSK occurs via physisorption. Investigation of the adsorption kinetics revealed the PSO and Elovich models best describe the sorption of either drug onto Ce–Py–MSK. The adsorption-desorption study revealed that 0.1 M HCl was the suitable eluent for RIFM from loaded Ce–Py–MSK with 94.25% desorption efficiency. The Ce–Py–MSK adsorbent was regenerated for five cycles with 82% RIFM removal efficiency, confirming the stability of the prepared adsorbent. Furthermore, the selectivity of the developed adsorbent towards RIFM and TIGC was confirmed compared to various interferents. Overall, the findings of this investigation show that the valorization of waste materials into green adsorbents is a promising, lucrative, and an environmentally friendly approach for depolluting pharmaceutical wastewater. Such an approach could be further extended to include the remediation of RIFM-TIGC binary mixtures both on the small and the large scale.

## Author contribution statement

Marwa El-Azazy: Conceived and designed the experiments; Analyzed and interpreted the data; Contributed reagents, materials, analysis tools or data; Wrote the paper.

Ahmed S. El-Shafie: Conceived and designed the experiments; Performed the experiments; Analyzed and interpreted the data; Wrote the paper.

Reem Al-Mulla, Siham S. Hassan: Performed the experiments; Wrote the paper.

Hassan I. Nimir: Analyzed and interpreted the data; Wrote the paper.

## Data availability statement

Data included in article/supp. Material/referenced in article.

## Funding

Open Access funding provided by the Qatar National Library.

## Declaration of competing interest

The authors declare that they have no known competing financial interests or personal relationships that could have appeared to influence the work reported in this paper. The authors declare the following financial interests/personal relationships which may be considered as potential competing interests.
